# A restatement of recent advances in the natural science evidence base concerning neonicotinoid insecticides and insect pollinators

**DOI:** 10.1098/rspb.2015.1821

**Published:** 2015-11-07

**Authors:** H. Charles J. Godfray, Tjeerd Blacquière, Linda M. Field, Rosemary S. Hails, Simon G. Potts, Nigel E. Raine, Adam J. Vanbergen, Angela R. McLean

**Affiliations:** 1 Oxford Martin School, c/o Department of Zoology, University of Oxford, South Parks Road, Oxford OX1 3PS, UK; 2 Plant Research International, Wageningen University and Research, PO Box 16, 6700 AA Wageningen, The Netherlands; 3 Rothamsted Research, Harpenden, Herts AL5 2JQ, UK; 4 NERC Centre for Ecology and Hydrology, Crowmarsh Gifford, Wallingford OX10 8BB, UK; 5 School of Agriculture, Policy and Development, University of Reading, Reading, UK; 6 School of Environmental Sciences, University of Guelph, Guelph, Ontario, Canada N1G 2W1; 7 NERC Centre for Ecology and Hydrology, Bush Estate, Penicuik, Edinburgh EH26 0QB, UK

**Keywords:** neonicotinoid, honeybee, bumblebee, pollinator, pest management, evidence for policy

## Abstract

A summary is provided of recent advances in the natural science evidence base concerning the effects of neonicotinoid insecticides on insect pollinators in a format (a ‘restatement') intended to be accessible to informed but not expert policymakers and stakeholders. Important new studies have been published since our recent review of this field (Godfray *et al.* 2014 *Proc. R. Soc. B* 281, 20140558. (doi:10.1098/rspb.2014.0558)) and the subject continues to be an area of very active research and high policy relevance.

## 1. Introduction

Neonicotinoid insecticides were introduced in the 1990s and their market share quickly expanded to approximately a third of the global insecticide total by value. They are used in different ways, but particularly as seed treatments where the chemical is absorbed by the growing plant and is distributed through all tissues at concentrations that can kill insect herbivores. However, neonicotinoids are also translocated to nectar and pollen where they can be consumed by pollinating insects. Numbers of pollinators have declined in agricultural landscapes and there is concern that the introduction and widespread use of neonicotinoids is partly responsible.

In December 2013, the European Union (EU) instigated partial restrictions on the use of neonicotinoid insecticides on crops that might be used as food by pollinating insects. This move is strongly opposed by many in the farming community and there has been a vigorous debate focusing on the scientific evidence that neonicotinoids harm pollinators, as well as the environmental and economic costs and benefits of the restrictions.

To try to assist the debate we produced a ‘restatement’ of the underlying natural science evidence base in a form that was intended to be accessible to informed but not expert policymakers and stakeholders [1]. Our avowed aim was to be as policy-neutral as possible while acknowledging that perfect neutrality is never achievable. The restatement was published as an appendix to a short paper in this journal accompanied by an extensive annotated bibliography as the electronic supplementary material.

Since the restatement was published the debate about restricting neonicotinoid use has continued unabated. Farming organizations have successfully applied for ‘120-day derogations’ from the restrictions in several European countries (see electronic supplementary material, paragraph A.2) on the grounds of lack of alternative pest-management options, moves that have been criticized by environmental non-governmental organizations. The EU is committed to review the restrictions in 2015–2016 and through the independent European Food Safety Authority opened a call for evidence (closing 30 September 2015; http://www.efsa.europa.eu/en/data/call/150522). Much new research has been published on the topic (we review over 80 studies here) including the largest replicated field study to date [2].

Despite the relatively short time since the restatement was published we provide here an update in the same format. We do this (i) because of the significant advances in the science; (ii) because of the continuing need for policy-neutral evidence summaries in this highly contested area, especially in the run up to the review of the EU restrictions; and (iii) in response to a request to do so by the UK Government Chief Scientific Adviser.

## 2. Methods

The literature on pollinators and neonicotinoids published since our restatement was completed was reviewed and a first draft evidence summary produced by a subset of the authors. All authors reviewed and revised the document, and agreed on the categorizing of the different evidence components using the same scheme we adopted earlier, and which is explained in paragraph A2 of the restatement update (appendix A). The second draft was sent to a series of stakeholders or stakeholder groups including scientists involved in pollinator research, representatives of the farming and agrochemical industries, non-governmental organizations concerned with the environment and conservation, and UK government departments and statutory bodies responsible for pollinator policy. The document was revised in the light of much helpful feedback from over 40 stakeholders (see acknowledgements). Though many groups were consulted, the project was conducted completely independent of any stakeholder and was funded by the Oxford Martin School (part of the University of Oxford).

## 3. Results

The update to the restatement of the natural science evidence base concerning neonicotinoid insecticides and insect pollinators is given in appendix A, with an annotated bibliography provided as the electronic supplementary material.

## 4. Discussion

The new evidence and evidence syntheses that have been published in the last 18 months (between February 2014 and August 2015) significantly advance our understanding of the effects of neonicotinoids on insect pollinators. Nevertheless, major gaps in our understanding remain, and different policy conclusions can be drawn depending on the weight one accords to important (but not definitive) science findings and the weightings given to the economic and other interests of different stakeholders. The natural science evidence base places constraints on policies that claim to be consistent with the science, but does not specify a single course of action.

We also raise an issue here that arises from our original study but is not directly relevant to the evidence base on the effects of neonicotinoids on pollinators. In introducing the subject we wrote ‘Neonicotinoid insecticides are a highly effective tool to reduce crop yield losses due to insect pests’, and in the restatement itself listed a small number of papers in the scientific literature to support this statement [1]. It has been pointed out that some of these papers were funded by industry and that there are other studies that have recorded no benefits of neonicotinoid use (e.g. [3]).

The efficacy of neonicotinoids is clearly an important issue, and we believe few would doubt that in some circumstances (combinations of crops, pests and locales) they are highly effective and in other circumstances they do not justify the costs of their purchase. We did not attempt to review this subject and should have been more careful to say we were not commenting on efficacy *per se*.

Though a meta-analysis of efficacy would be very informative it would also be very difficult. Efficacy studies are largely conducted by industry, the sector that benefits most from the data, and are not the type of science usually funded by public organizations. Typically, the studies are not published in the peer-reviewed literature (though they are often made available to regulators) and some are kept confidential for commercial reasons. Efficacy trials are expensive and it seems unlikely that they will ever be publicly funded at scale. It is an interesting topic for debate whether industry would benefit in the long run from placing more of its data in the public domain as well as putting in place measures to increase public confidence in studies they fund themselves. The recent movement in the pharmaceutical sector to set up trial registries (see https://clinicaltrials.gov/ct2/home and https://www.clinicaltrialsregister.eu) provides a model for how the latter might be achieved.

## Supplementary Material

Annotated Bibliography

## Appendix A. ‘A restatement of recent advances in the natural science evidence base concerning neonicotinoid insecticides and insect pollinators'

For an annotated bibliography of the evidence supporting each statement (hereafter ‘Annotated Bibliography’) see the electronic supplementary material.

**(a) Introduction and aims**

A1 This document is an update to our previous ‘restatement' of the natural science evidence base concerning neonicotinoid insecticides and insect pollinators. It does not repeat evidence presented earlier and concentrates on material published between February 2014 and August 2015. It is arranged in the same six sections (a–g). Paragraphs are numbered A1, A2, etc. and the symbol § (e.g. §16) is used to indicate the paragraph number in the original document [1], where the same subject was treated.
A2 (§1) The restrictions on the use of certain neonicotinoids as seed coatings on crops attractive to pollinating bees will have been in place for two years in December 2015. The Commission has now mandated the European Food Safety Authority to collate relevant data as the first step in the review of these measures. Industry groups in a number of EU countries have successfully applied for ‘120-day' derogations to use restricted neonicotinoids in defined geographical areas on the grounds of the absence of viable alternatives (see also A33). The province of Ontario in Canada is introducing restrictions on neonicotinoid use on maize (corn) and soy from July 2015. We are not aware of other equivalent measures that have been introduced elsewhere in the world.
A3 (§2) As before the authors provide a consensus judgement on the nature of the different evidence components. We use the following descriptions, which explicitly are not a ranking, indicated by abbreviated codes. Statements are considered to be supported by:
  [D_ata_] A strong evidence base involving experimental studies or field *data* collection, with appropriate detailed statistical or other quantitative analysis.
  [E_xp_op_] A consensus of *expert opinion* extrapolating results from related ecological systems and well-established ecological principles.
  [S_upp_ev_] Some *supporting evidence* but further work would improve the evidence base substantially.
  [P_rojns_] *Projections* based on the available evidence for which substantial uncertainty often exists that could affect outcomes.

**(b) Pollinators and neonicotinoid insecticides**

A4 (§§4–11) In the Annotated Bibliography we list new references relevant to the introductory material in this section.

**(c) Exposure of pollinators to neonicotinoid insecticides**

A5 (§§13–14) As in the first version of the restatement we consider concentrations of neonicotinoids in pollen and nectar of the order of 2–6 ng g^−1^ to be typical of those that a pollinator might encounter when foraging on seed-treated crops. Statements about low or high concentrations are made relative to this benchmark, though we acknowledge there will be variation around these figures and that this benchmark involves an element of expert judgment. A wide-ranging review of how neonicotinoids, introduced as seed coatings, may move through and persist in the environment has been published. [E_xp_op_]
A6 (§15) There is evidence that contaminated dust expelled into the environment from drilling machines during the planting of seeds treated with neonicotinoids can continue to pose threats to honeybees. [D_ata_]
A7 (§16) There continues to be intensive study of movement of neonicotinoids through the environment and their effect on non-pollinating organisms. This topic is outside the scope of this restatement though in the Annotated Bibliography we provide an entry into this literature. [E_xp_op_]
A8 (§18) A laboratory study of honeybee and bumblebee (*Bombus terrestris*) behaviour showed that foraging-age insects do not avoid food sources containing imidacloprid, thiamethoxam or clothianidin at field relevant concentrations (approx. 0.25–3 ng g^−1^). The bees do not seem able to ‘taste' these compounds though there is evidence that the first two stimulate feeding. The response is affected by insect age: newly emerged honeybees and bumblebees largely avoid imidacloprid-contaminated sugar solution. [D_ata_] These results suggest that it may be less likely that individual flower-visiting bees will reduce their pesticide exposure by avoiding flowers in the field contaminated by insecticides, but this needs to be tested in the field. [E_xp_op_]
A9 (§20) Honeybee colonies placed in or beside fields of flowering oilseed rape (canola) forage extensively on the crop, though those situated further away may use it much less, even in landscapes where it is the dominant bee-attractive crop. There is limited evidence for similar patterns in other bee species. [D_ata_]
A10 (§21) *Summary.* Some information is available on the extent to which pollinators are exposed to neonicotinoids through different pathways in the environment. Most exposure will be at sublethal levels from foraging on seed-treated plants, the most important exception being contamination from dust at the time of planting, especially when regulations and best practice are not followed. Better quantitative data on typical concentrations in nectar and pollen of non-crop plants in agricultural landscapes and the extent of exposure through planting dust and other sources is desirable, as is improved data on how different species of pollinating bees collect food in different landscapes. [E_xp_op_]

**(d) Laboratory studies of lethal and sublethal effects of neonicotinoids**

A11 (§§22–27) New reviews of the literature on lethal and sublethal effects of neonicotinoids on pollinators, and a large literature survey, have been published. [E_xp_op_]
A12 (§25) Further studies have shown the potential of neonicotinoids to cause detrimental sublethal effects in different species of flower-visiting bees, as well as the complexity of the physiological response of larval and adult honeybees to acute and chronic sublethal neonicotinoid exposure. How sublethal doses of neonicotinoids affect behavioural processes such as homing ability in honeybees is strongly context-dependent (affected by, for example, temperature and landscape structure) complicating the design of standard assays of sublethal effects. Recent studies have associated chronic low doses of neonicotinoids with neuronal dysfunction in the brain of bumblebees and increased vulnerability to other neural stressors. [D_ata_]
A13 (§26) There is some new evidence that biological and non-biological stresses can exacerbate sublethal effects of neonicotinoids, though such effects are not universal and are difficult to predict. [S_upp_ev_]
A14 (§27a) A new survey of toxicity data shows that the relative sensitivity to different pesticides of honeybees and other pollinating bees is highly variable [D_ata_], which limits the degree to which honeybee data can be extrapolated to other sentinel species. [E_xp_op_]
A15 (§28) *Summary.* Data continue to accumulate showing that sublethal neonicotinoid exposure can affect many aspects of pollinator behaviour and physiology (though most studies involve honeybees or bumblebees). Sublethal effects at field-realistic doses are now established, but their consequences for pollinator populations and pollination are still unclear. Responses to neonicotinoids vary across bee species and are affected by type of exposure (for example, acute versus chronic or oral versus contact), which makes generalisations difficult. [E_xp_op_]

**(e) Neonicotinoid residues observed in pollen, nectar and wax in the field**

A16 (§§29–31) New data, data compilations and reanalyses of earlier data continue to show that neonicotinoid residues can be detected in pollen and nectar collected by pollinating bees. However, these data are highly variable, making general inference hard. [S_upp_ev_] Incidences of high neonicotinoid residues that would almost certainly cause acute toxic effects in honeybees and bumblebees do occur, but not commonly. [E_xp_op_]
A17 (§32) *Summary* (*unchanged from earlier restatement*). Neonicotinoids can be detected in wild pollinators as well as honeybee and bumblebee colonies but data are relatively few and restricted to a limited number of species. Studies to date have found low levels of residues in surveys of honeybees and honeybee products. Observed residues in pollinating bees and the products they collect will depend critically on details of spatial and temporal sampling relative to crop treatment and flowering. [E_xp_op_]

**(f) Experiments conducted in the field**

A18 (§33) As before, we give separate, detailed treatment to ‘semi-field' studies where insects are exposed by the experimenter to a known dose of insecticide and then allowed to forage in the environment, and ‘true field’ studies involving exposure to neonicotinoids as applied in actual farm landscapes. There is continuing debate about the relevance of the doses and application methods used in semi-field studies, and about the relevance of methodologies used in true field experiments. [E_xp_op_]
A19 *Dively et al.* [4] provided replicate colonies of honeybees over a 12-week period with supplemental pollen paste diets containing imidacloprid at three concentrations (5, 20 and 100 ng g^−1^) with a fourth control treatment. Experiments were conducted in 2009 (10 replicates per treatment) and 2010 (seven replicates). They found no effect on foraging performance or colony health in the short term but over a longer period, colonies exposed to neonicotinoids were more likely to lose queens, suffer higher overwintering mortality and have greater *Varroa* infestations, though these effects were only statistically significant at the high (20) and very high (100 ng g^−1^) concentrations. [D_ata_] The authors concluded that their results did not suggest that neonicotinoids were a sole cause of colony collapse. [P_rojns_]
A20 *Lu et al.* [5]*.* Honeybee colonies were fed with syrup containing high concentrations of imidacloprid or clothianidin, or with no added insecticide, for a 13-week period from July to September (in Massachusetts, USA). A detrimental effect of neonicotinoids on successful overwintering was reported though we have concerns (see Annotated Bibliography) about how this conclusion was reached. [E_xp_op_]
A21 (§37) *Gill & Raine* [6] reported how the day-to-day foraging patterns of 259 bumblebee (*B. terrestris*) workers from 40 colonies were affected by individual or combined exposure to the neonicotinoid imidacloprid and the pyrethroid *λ*-cyhalothrin. These data, and results presented by *Gill et al.* [7], were collected in the same experiment conducted in 2011 (see §37). Exposure to imidacloprid concentrations (10 ng g^−1^) towards the high end of what is typically observed in the field led to acute and chronic effects on individual foraging behaviour (although actual imidacloprid consumption by individual workers will have been diluted by foraging from untreated floral sources in the field; see §37). Whereas individual bumblebee foraging efficiency normally improves with experience, this did not occur in individuals exposed to imidacloprid. [D_ata_] Evidence was found that the insecticide affected the pollinators' preference for different flowers as sources of pollen. [S_upp_ev_]
A22 *Moffat et al.* [8]. Bumblebee (*B. terrestris*) colonies were provided with syrup containing low doses (approx. 2 ng g^−1^) of imidacloprid and placed in the field in a non-intensive agricultural location for 43–48 days. By most measures, the neonicotinoid had a significantly negative effect on colony performance compared with controls. [D_ata_]
A23 (§38) A true field experiment by Thompson *et al.* [9] was originally interpreted as showing no effects of two neonicotinoids on bumblebee (*B. terrestris*) colony performance. The experiment placed multiple colonies adjacent to oilseed rape fields that had received different insecticide treatments (but with no replication at the field level). A colony-level reanalysis of the data by Goulson [10] showed a significant relationship between neonicotinoid concentration and performance: colonies with higher concentrations of thiamethoxam or clothianidin in nectar, or thiamethoxam in pollen stores, produced significantly fewer new queens. Because exposure was not manipulated at the colony level, this study should be considered as correlational rather than experimental. [P_rojns_]
A24 *Cutler et al.* [11]. Ten 2-hectare plots in Southern Ontario, Canada, were planted with oilseed rape, half of which were planted with seed treated with the neonicotinoid clothianidin with the other half controls. During peak flowering, four honeybee hives were placed in the centre of each field for two weeks before being moved to a site away from insecticide treated crops. Pollen from hives in treated fields had higher concentrations of clothianidin (which were non-zero in controls) but no effects of the insecticide were found for a variety of honeybee colony growth or overwintering metrics. [D_ata_]
A25 *Cutler & Scott-Dupree* [12]*.* Bumblebee (*Bombus impatiens*) colonies were placed beside four fields planted with organic maize and four with maize grown from neonicotinoid-coated seeds in Southern Ontario, Canada. The study took place on commercial farms and organic and non-organic maize produced pollen at different times. No differences were found in ten measures of colony health, except that colonies by treated fields had significantly fewer workers (which the authors attributed to an effect of crop development time). Analysis of collected pollen showed maize was a very small component (0–2%) of these bumblebees' diets. [D_ata_]
A26 *Rundlöf et al.* [2]. In southern Sweden eight pairs of spring-sown oilseed rape fields were chosen with one of each pair grown from clothianidin coated seeds and the other from non-coated seeds. The seed treatment used, as recommended by the manufacturer, led to higher concentrations of clothianidin in pollen than is normally observed in this crop. Treated fields had lower densities of solitary bees and bumblebees, and poorer bumblebee (*B. terrestris*) colony growth and queen production (all comparisons statistically significant). Solitary bees (*Osmia bicornis*) placed adjacent to treated fields all disappeared while a small but significantly higher number nested beside control fields. The experiment detected no significant effects on measures of honeybee colony strength. Wildflowers, to which pollinators may also be exposed, had higher levels of clothianidin when growing in uncultivated land around treated compared to untreated crops. [D_ata_]
A27 (§40) *Summary.* Evidence continues to accumulate from semi-field experiments that sublethal exposure to neonicotinoid insecticides, chiefly but not exclusively at the high end of what is likely to be experienced in the environment, can affect foraging and other behaviours in the field. Several true field studies have reported no effect of exposure to neonicotinoid-treated crops on honeybee colony performance, but the first large-scale study of the exposure of bumblebees (see A26) found strong evidence of harmful effects. There is very little information about the effects of neonicotinoids on non-bee pollinators. [E_xp_op_]

**(g) Consequences of neonicotinoid use**

A28 (§41) A new, open access computer model of honeybee colony performance has been developed that could help integrate the effects of different stressors (including insecticide exposure on colony performance). Models of the effects of sublethal stress, including insecticide exposure, on bumblebee colony dynamics and failure rates have also been developed. [E_xp_op_]
A29 *Budge et al.* [13] collected data on honeybee colony in-season loss and neonicotinoid use from nine regions of the UK every other year from 2000 to 2010. Controlling for region (but not year) they find a weak but significant correlation between colony loss and imidacloprid use, but not total neonicotinoid use. We found that this effect was due to a correlation between annual average colony loss and imidacloprid use. Imidacloprid use peaked mid-decade (after which it was replaced by thiamexotham and clothianidin) and there was a tendency for honeybee losses to be higher at this time. Because other factors not included in the analysis may show similar annual patterns, and because of statistical issues with the analysis (see Annotated Bibliography), the correlation of honeybee colony losses with imidacloprid use, and the lack of correlation with total neonicotinoid use, should be treated with great caution. [E_xp_op_]
A30 (§42) A meta-analysis suggests that 80% of the pollination of global crops for which wild bees are responsible can be attributed to the activities of just 2% of species. These also tend to be species that are most responsive to interventions designed to increase bee densities. [E_xp_op_] The most important species of wild bees in Europe and North America are common species of bumblebee (*Bombus* spp.) underlying the importance of understanding their interaction with insecticides. [E_xp_op_]
A31 (§43) Evidence continues to accumulate on the drivers of pollinator decline. Analyses of the extinction rates (since 1850) and changes (1921–1950 versus 1983–2012) in species richness and composition of bees and wasps in the UK suggests land use and management changes are the most important historical drivers with major faunal losses occurring early in the twentieth century. Any effects of changes in pesticide use over recent decades are unlikely to be picked up by these analyses. An analysis of the historical shifts in the ranges of European and North American bumblebees showed that they have failed to track climate warming at their northern range limits, while southern range limits have contracted. These shifts were independent of changes in land use (both continents) and pesticides application, including neonicotinoids (in North America only; pesticide data was unavailable for Europe). This study only assessed changes in species range distributions, and so any impacts of pesticides on population density or diversity at finer habitat or landscape scales would not be identified. [S_upp_ev_]
A32 (§44) Updates on overwintering honeybee colony loss in Europe and North America (USA and Canada) have been published. [D_ata_]
A33 (§45) There are still few data examining the effects of the neonicotinoid restrictions on pest numbers and consequently on crop yields and income, on farmers' decisions about whether to grow crops subject to restriction, or on alternative pest-management strategies used by farmers. A recently published study suggests farmers that use neonicotinoid seed treatments on oilseed rape in the UK use fewer subsequent foliar insecticide applications in the autumn but with no overall effect on applications at flowering time. [E_xp_op_]
A34 (§46) *Summary*. There still remain major gaps in our understanding of how pollinator colony-level (for social bees) and population processes may dampen or amplify the lethal or sublethal effects of neonicotinoid exposure and their effects on pollination services; as well as how farmers might change their agronomic practices in response to restrictions on neonicotinoid use and the resulting positive or negative effects on pollinators and pollination. While these areas continue to be researched there is still a limited evidence base to guide policymakers on how pollinator populations will be affected by neonicotinoid use or how agriculture will respond to neonicotinoid usage restrictions*.* [E_xp_op_]
