# Supplementary material for: A restatement of recent advances in the natural science evidence base concerning neonicotinoid insecticides and insect pollinators
Source: Proc Biol Sci. 2015 Nov 7;282(1818):20151821. doi: 10.1098/rspb.2015.1821 (PMC4650156; doi:10.1098/rspb.2015.1821)
Supplement: Annotated Bibliography [file rspb20151821supp1.pdf]

# Annotated bibliography to accompany:

# A restatement of recent advances in the natural science evidence base concerning neonicotinoid insecticides and insect pollinators

Proc. R. Soc. B 20151821; DOI: 10.1098/rspb.2015.1821

H. Charles J. Godfray, Tjeerd Blacquière, Linda M. Field, Rosemary S. Hails, Simon G. Potts, Nigel E. Raine, Adam J. Vanbergen & Angela R. McLean

Paragraph numbering corresponds to that in the main document; full references at end. The symbol § (for example §16) is used to indicate the paragraph number in Godfray *et al.* (2014) where the same subject was treated. Website URLs were accessed 10 September 2015.

## (a) Introduction and aims

(A.1) This document updates Godfray *et al.* (2014).

(A.2) (§1) European Union policy on pollinators and neonicotinoids is described at http://ec.europa.eu/food/animals/live_animals/bees/pesticides/index_en.htm. Judging by press reports, 120-day derogations have been granted by a number of countries (including for oilseed rape (canola) in Finland, Denmark and Estonia and for maize in Romania, Bulgaria & Serbia) and rejected or withdrawn in others (including Germany and until recently the UK). The UK government approved a limited derogation in July 2015 (http://www.nfuonline.com /news/latest-news/nfu-comment-on-neonicotinoid-distribution), but full details are not yet available. We are not aware of a database listing successful and unsuccessful derogation applications. From July 2015 a policy aimed to significantly reduce usage of neonicotinoid seed coatings on maize (corn) and soy will come into effect in the province of Ontario (Canada) (http://www.ontario.ca/environment-and-energy/neonicotinoid-regulations). The stated aim is to "reduce the number of acres planted with neonicotinoid-treated corn and soybean seed by 80 per cent by 2017" [from 2015 levels]. Farmers seeking to use neonicotinoid treated seed for these crops will need to provide evidence of a demonstrated pest problem.

(A.3) (§2) Unchanged from Godfray *et al.* (2014).

## (b) Pollinators and neonicotinoid insecticides

(A.4) (§4-§11) Simon-Delso *et al.* (2015) provide an extensive review of the history of neonicotinoids, their discovery, mode of action, metabolism (in animals, plants and the environment) and their growing share of the insecticide market. Casida and Durkin (2013) is a general review of neuroactive insecticides. There have also been recent studies of the effect on pollinators of using neonicotinoid sprays on insect pests of turf grass Larson *et al.* (2013), Larson *et al.* (2014), Larson *et al.* (2015). Shao *et al.* (2013) review the development and application of neonicotinoids in China.

## (c) Exposure of pollinators to neonicotinoid insecticides

(A.5) (§13-§14) Our estimates for benchmark concentrations of neonicotinoids that pollinating bees are likely to encounter when foraging on crops grown from treated seeds (but not contaminated by dust) are based on Blacquière *et al.* (2012). The European Food Safety Authority comes to similar conclusions using a different methodology (considering different recommended field application rates) for example for imidacloprid in oilseed rape they estimate 1.59 - 8.35 ng $g^{-1}$ in nectar and 1.56 - 8.19 ng $g^{-1}$ in pollen European Food Safety Authority (2013b, p.18) see also European Food Safety Authority (2013c), European Food Safety Authority (2013a). Bonmatin *et al.* (2015) describe in detail the uptake and movement of neonicotinoids through plants, including the process of guttation (exudation of xylem fluid from the leaves of some plants), and tabulate residue levels in pollen and nectar from different treated crops. They find great variability in range, means and maximum doses (though some of their high figures involve contamination through dust, see next paragraph).

(A.6) (§15) Exposure of pollinators and other non-target organisms to contaminated dust from seed-planting machines is reviewed by Bonmatin *et al.* (2015) and by Krupke and Long (2015); despite attention by regulators they consider it a likely cause of environmental contamination, in particular when best practice is not followed. Cutler *et al.* (2014a) discuss recent incidents of bee mortality involving contaminated dust (chiefly from planting maize) in Canada (also summarized by Health Canada (2014)) though they note that these are declining in number. Stewart *et al.* (2014) found cases of neonicotinoids in the tissues of wildflowers around fields in the mid-southern USA immediately after planting (with maize, cotton & soybean) that they attribute to contaminated dust (23% samples positive; average [maximum] 10 [257] ng $g^{-1}$). Rundlöf *et al.* (2015), in an experiment described more fully in paragraph (A.26), find plants in margins around fields with neonicotinoid seed-treated oilseed rape had higher levels of clothianidin (1.2 ± 0.8 v 0 ng $g^{-1}$) in flowers and leaves immediately after sowing compared with untreated crops. The levels in wildflowers around treated crops remained approximately the same two weeks after sowing though at that time measurements were not taken around untreated crops. See also Heimbach *et al.* (2014); Samson-Robert *et al.* (2014).

(A.7) (§16) Bonmatin *et al.* (2015) review the movement of neonicotinoids through soil and water bodies, and toxicity to soil and aquatic invertebrates; see also de Perre *et al.* (2015), Jones *et al.* (2014), Schaafsma *et al.* (2015). Pisa *et al.* (2015) include an extensive review of the effects of neonicotinoids on non-target invertebrates. Barbieri *et al.* (2013) show differential sublethal effects on non-target arthropods (ants) and propose that neonicotinoids could affect community structure. Douglas *et al.* (2015) found evidence that slugs eating soya beans grown from seeds coated with thiamethoxam contained sufficient neonicotinoid to harm the predatory beetles that fed on them, so impairing biological control, and Frewin *et al.* (2014) found

negative effects of neonicotinoids on soya bean aphid parasitoids. Different effects in the aquatic environment are discussed by Anderson *et al.* (2015), Colombo *et al.* (2013), Daam *et al.* (2013), Hayasaka *et al.* (2013), Main *et al.* (2014), Morrissey *et al.* (2015), Pisa *et al.* (2015), Smit *et al.* (2015), van Dijk *et al.* (2013), Vijver and van den Brink (2014). Gibbons *et al.* (2015) review the effects of neonicotinoids on wild vertebrates, while Hallmann *et al.* (2014) argue that neonicotinoids are indirectly responsible for reductions in the density of insectivorous birds in Holland. For a debate over the interpretation of such imidacloprid aquatic toxicity data in the Netherlands see van Dijk *et al.* (2013) and Vijver and van den Brink (2014).

(A.8) (§18) The study of bee behavioural responses to neonicotinoids was by Kessler *et al.* (2015), see also the commentary on this work by Raine and Gill (2015). For both honeybees and bumblebees thiamexotham and imidacloprid stimulated increased relative consumption of contaminated food sources at field realistic doses. This was despite imidacloprid consistently reducing total consumption in bumblebees (thiamexotham and clothianidin reduced total consumption in bumblebees and honeybees only at the higher concentrations). In a different study, Easton and Goulson (2013) found that very low doses (1 ng $g^{-1}$) of imidacloprid in insect traps (yellow pan traps) led to lower catches of pollinating flies and beetles, though how this observation relates to pollinators foraging in the field is not clear.

(A.9) (§20) Rundlöf *et al.* (2015) placed colonies or nests of bees adjacent to oilseed rape fields in Sweden (see also (A.26)) and found this plant species composed 53-63% of pollen collected by honeybees, 75-88% by bumblebees (*Bombus terrestris*), and 35% for a solitary bee (*Osmia bicornis*). Cutler *et al.* (2014b) working in Southern Ontario found 88% of pollen loads at some times to be composed of oilseed rape pollen when honeybee colonies were placed in the middle of fields. Garbuzov *et al.* (2015) decoded honeybee waggle dances to deduce limited foraging of rural bees on oilseed rape in southern England (most foraging was within 2 km of the hive), a conclusion supported by pollen sampling (oilseed rape constituted up to 14% by weight). Some data on other bee species in Holzschuh *et al.* (2013). Stanley *et al.* (2013) and Stanley and Stout (2014) regularly found three species of bumblebees (*B. terrestris*, *B. lucorum* and *B. cryptarum*) foraging in oilseed rape fields in Ireland. Overall 35% of all flower visits to oilseed rape were made by bumblebee species, 32% from hoverflies, 17% from honeybees, 12% from other flies and about 2.5% from solitary bees.

(A.10) (§21) Authors.

## (d) Laboratory studies of lethal and sublethal effects of neonicotinoids

(A.11) (§22-§27) Review by Pisa *et al.* (2015); see also Rondeau *et al.* (2014) on chronic exposure. Fairbrother *et al.* (2014) review the risks to honeybees of neonicotinoids and Johnson (2015) is a general review of honeybee toxicology paying particular attention to the spectrum of compounds and combination of compounds that the insects are exposed to. Tome *et al.* (2015) assess imidacloprid toxicity in a species of meliponine (stingless) bee, an important but under-studied group of tropical pollinators. Fryday *et al.* (2015) provide an exhaustive annotated list of publications discussing adverse effects on pollinating bees of imidacloprid, clothianidin and thiamexotham. Lundin *et al.* (2015) survey studies of the effects of neonicotinoid on bees pointing out their geographical and taxonomic biases [this study was published while our work was in press and hence is only briefly mentioned here].

(A.12) (§25) In the laboratory neonicotinoids increase acetylcholinesterase activity and Boily *et al.* (2013) found that honeybees from hives placed next to conventional crops had higher activity of this enzyme than those located by organic crops or uncultivated ground, though whether neonicotinoids were responsible could not be established. Hatjina *et al.* (2013) found that newly emerged honeybees fed food contaminated with low doses of imidacloprid (at 2-3 ng $g^{-1}$) developed smaller hypopharyngeal glands and had perturbed patterns of abdominal ventilation ("breathing"), while cytological effects of sublethal exposure to thiamethoxam were studied by Oliveira *et al.* (2014). Henry *et al.* (2014) showed that relatively high, but sublethal, doses of neonicotinoids have effects on honeybee foraging in natural environments that are influenced by weather and landscape structure. Fischer *et al.* (2014) observed impaired honeybee navigation after insects had received high sublethal oral doses (per bee) of 2.5 ng clothianidin or 7.5 & 11.5 ng imidacloprid. Sandrock *et al.* (2014b) found that field-realistic sublethal doses of thiamethoxam and clothianidin reduced short-term honeybee colony performance, but colonies recovered and there was no effect on overwintering fitness. However, impaired colony growth associated with queen failure and reduced swarming was observed in the following spring (an effect of honeybee genetic background was also observed). Exposing honeybees to contaminated food solutions (10ng $g^{-1}$ imidacloprid, thiamethoxam or clothianidin for 24hr) affected some but not all motor functions Williamson *et al.* (2014). Asian (or Eastern) honeybees (*Apis cerana*) fed relatively high levels of imidacloprid (34 ng $g^{-1}$), showed poorer predator avoidance and reduced foraging performance Tan *et al.* (2014). The same species fed much lower doses of imidacloprid (0.1 ng per bee) as adults showed impaired olfactory learning, as did adults developing from larvae exposed to doses of 0.24 ng per individual (there was no effect on mortality) Tan *et al.* (2015). Elston *et al.* (2013) exposed queenless microcolonies of bumblebees (*B. terrestris*) to supplementary diets containing low (1 ng $g^{-1}$) or high (10 ng $g^{-1}$) concentrations of thiamethoxam (plus a control). Fewer wax cells were produced in both treatments and no larvae were produced at the higher exposure. Scholer and Krischik (2014) provided bumblebee colonies (*B. impatiens*) in greenhouses with relatively high doses (≥ 10ng $g^{-1}$) of imidacloprid and clothianidin and observed a variety of sublethal and lethal consequences. Moffat *et al.* (2015) found that chronic field-realistic low doses (~2 ng $g^{-1}$) of clothianidin and imidacloprid accumulated in the brains of bumblebees (*B. terrestris*) and led to mitochondrial dysfunction in neurons (that was exacerbated by the presence of acetylcholine). This provides a mechanism that may explain the effects of neonicotinoids on navigation and raises the possibility that actively foraging insects (in which higher levels of acetylcholine will be present) may have greater susceptibility to sublethal exposure (see also (A.22)). Chronic sublethal dietary exposure of a solitary bee (*O. bicornis*) to low doses of thiamethoxam (2.87 ng $g^{-1}$) and clothianidin (0.45 ng $g^{-1}$) led to large reductions in lifetime reproductive success Sandrock *et al.* (2014a).

(A.13) (§26) Archer *et al.* (2014) found protein-fed honeybees better able to withstand nicotine stress (which the authors interpret as implying stressed bees may be more susceptible to neonicotinoids). Doublet *et al.* (2015) and Retschnig *et al.* (2014) found that thiacloprid (a neonicotinoid, though not one used frequently as a seed treatment) elevated larval mortality due to

Black Queen Cell Virus and adult mortality due to *Nosema ceranae* (a microsporidian parasite) respectively. Fauser-Misslin *et al.* (2014) fed laboratory colonies of bumblebees syrup contaminated with low doses of thiamethoxam (4 ng $g^{-1}$) and clothianidin (1 ng $g^{-1}$) over a nine-week period, a relatively long period of chronic exposure. The treatments were crossed with exposure to the bumblebee pathogen *Crithidia bombi.* Reductions in worker longevity and colony productivity were observed in the neonicotinoid treatments, but they were not exacerbated by the presence of the pathogen. In contrast, queen longevity was significantly reduced only in the combined neonicotinoid and pathogen treatment. Results from a similar crossed design study, using a pyrethroid insecticide (λ-cyhalothrin) and the same parasite (*C. bombi*), found no effect of insecticide exposure on worker longevity, susceptibility to parasitic infections or colony development, but did lead to a reduction in worker body mass Baron *et al.* (2014). Thompson *et al.* (2014) studied synergistic effects of neonicotinoids and routinely used fungicides. Using field realistic (but worst case) doses of four fungicides (flusilazole, propiconazole, tebuconazole and myclobutanil) the impact on the $LD_{50}$ of four neonicotinoids (thiamethoxam, clothianidin, imidacloprid and thiacloprid) was examined via contact and oral doses. Significant (though not testing for multiple comparisons) synergistic effects on $LD_{50}$ were found in two of the 32 combinations tested (contact doses of thiamethoxam with tebuconazole and oral doses of clothianidin with tebuconazole). In a field experiment Moffat *et al.* (2015) (see (A.22)) found no significant synergistic effects of the organophosphate chlorpyrifos and imidacloprid.

(A.14) (§27a) Arena and Sgolastra (2014) compared the $LD_{50}$s of a very broad range of pesticides including imidacloprid (ten studies) and thiamethoxam (four studies) on different bee species relative to the honeybee. For imidacloprid there was a wide range of relative susceptibilities (approximately equal numbers of studies found the honeybee to be more or less susceptible than other bee taxa) while the fewer studies of thiamethoxam all found honeybees had higher $LD_{50}$s (i.e. were less susceptible).

(A.15) (§28) Authors.

## (e) Neonicotinoid residues observed in pollen, nectar & wax in the field

(A.16) (§29-§31) Bonmatin *et al.* (2015) reviewed translocation of neonicotinoids to nectar and pollen and the likely concentrations there. They provide tables of estimates of neonicotinoid incidence and concentrations from studies we previously reviewed plus some new material. See also Krupke and Long (2015). Sanchez-Bayo and Goka (2014) reviewed data for the concentration of pesticides in nectar, pollen and wax. They develop an index of risk based on (i) frequency of presence for residues; (ii) average and maximum concentrations when present; and (iii) published toxicity through different exposure regimes (contact or ingestion, acute or chronic). Using heterogeneous data from all geographical regions they conclude that clothianidin, imidacloprid and thiamethoxam rank highly in their potential risks to honeybees and bumblebees compared to other pesticides. See also Kasiotis *et al.* (2014). Pohorecka *et al.* (2013) found high levels of clothianidin in pollen collected by honeybees from hives placed next to maize fields in Polish agricultural landscapes irrespective of the insecticide regime applied to the crop. Fryday *et al.* (2015) provide an exhaustive annotated list of publications relevant to exposure of pollinating bees to imidacloprid, clothianidin and thiamexotham.

(A.17) (§32) Authors.

## (f) Experiments conducted in the field

(A.18) (§33) Issues concerning semi-field experiments chiefly revolve around whether the dose and method of application is relevant to the experience of pollinators in the field. Thus Carreck and Ratnieks (2014) critique the doses used in several of the semi-field experiments, arguing that they are probably higher than bees are likely to experience foraging in farmed landscapes. Issues around field experiments include the length of time bees are exposed to neonicotinoid-treated crops, and what happens to colonies after exposure: for example the realism of removing them to habitats with no risk of further insecticide exposure and the extent to which they are given additional food. In addition, the controls used in field experiments are usually the absence of all insecticides and not the alternative pest management strategy that would be adopted by a farmer. A no-insecticide control tests the absolute effect of neonicotinoids while an alternative pest-management control tests the effect of neonicotinoid restrictions. After this article was in press a study on honeybee colonies around two treated and two control fields in Canada was published by Alburaki *et al.* (2015).

(A.19) Dively *et al.* (2015).

(A.20) Lu *et al.* (2014) estimated that each bee in the neonicotinoid treatment consumed 0.74 ng of insecticide per day. Replication was six colonies per treatment; three of these colonies received syrup made from sucrose mixed with water, the other three colonies were fed high-fructose corn syrup over the study period. The number of frames within each hive occupied by bees was monitored over the subsequent winter. Five out of six control colonies and either two (imidacloprid) or four (clothianidin) out of six colonies in the neonicotinoid treatments survived. From mid-winter control hives were reported to have significantly more occupied frames than insecticide treated hives. The quoted average consumption of neonicotinoid insecticides is 3-12 times higher than the worst-case nectar ingestion rates calculated in §22e; the assumption of a constant 50,000 bees per colony is high and were it lower the consumption rate would be even greater. Foraging bees, which consume more food, will also experience higher exposure. We were not able to understand the statistical analysis from the description in the paper and after discussion with the authors requested sight of the original data (May 28th 2014) which has not yet been granted.

(A.21) Gill and Raine (2014) reported new analyses of the semi-field experiment on 40 bumblebee colonies reported in Gill *et al.* (2012); see §37 for discussion of insecticide application technique and relevance to field concentrations.

(A.22) Moffat *et al.* (2015) found significant effects of imidacloprid on the number of live bees and healthy brood cells, the total bee mass in the nest and the final mass of the nest, but not on the average mass of live bees. The imidacloprid treatment was crossed with a second treatment involving the potential antagonist chlorpyrifos (an organophosphate), but this had no statistically significant extra effects on colony performance. Six colonies (placed three to a box) were assigned to each of the four treatments. The paper also reported studies of the physiological effects of low (~2ng $g^{-1}$) concentrations of imidacloprid on bumblebee brain function (see (A.12)).

(A.23) (§38) Reanalysis of Thompson et al (2013; http://fera.co.uk/ccss/documents/defraBumbleBeeReportPS2371V4a.pdf) by Goulson (2015) which differed in statistical methodology, treatment of low concentration data values, and

exclusion of points considered outliers. Commenting on the experiment in §38a we said "the experimental design, in particular the lack of replication at field level and absence of a clear effect of treatment, allows only limited inference about the effects of neonicotinoids in the field" which we consider still applies.

(A.24) The plots used by Cutler *et al.* (2014b) were small (2 ha) compared to most commercial fields, and the potential pesticide exposure period was less than likely to occur under typical conditions. Most (88%) pollen collected by the honeybees in the first week was oilseed rape but in the second week this fell to 46%. For each colony they measured colony weight gain, pollen collected, honey yield, number of adults and number of sealed brood cells, as well as *Varroa* mites per bee, *Nosema* infection and number of dead bees. Colonies, especially at first, foraged predominantly on the spring-sown oilseed rape (88% in first week, 46% in second) where exposure in pollen was estimated as ~0.5-2 ng $g^{-1}$. Clothianidin concentrations in pollen collected by bees in treated fields was low, 0.84 ± 0.49 ng $g^{-1}$, and in control fields three-times less but non-zero at 0.24 ± 0.44 ng $g^{-1}$ (an analysis that forced confidence limits to be non-negative would have been preferable). Overwintering success and a range of other endpoint measures did not significantly differ between treated and control hives. A statistical power analysis of the experiment was not reported.

(A.25) The Cutler and Scott-Dupree (2014) study used fields planted by commercial farmers and hence was not strictly a controlled experiment and some potentially confounding factors were noted. For example, the potential pesticide exposure period was less than likely to occur under typical conditions,. For example, the neonicotinoid seed-treated maize produced pollen earlier than the organic maize, and different neonicotinoid (involving clothianidin and/or thiamethoxam) treatments were applied in the four non-organic treatments. Neonicotinoid levels reported in maize pollen (0.1 – 0.8 ng $g^{-1}$ in treated fields) are low compared to typical concentrations. Colonies were moved after 5-6 days to a neonicotinoid free environment. A statistical power analysis of the experiment was not reported.

(A.26) The Rundlöf *et al.* (2015) paper in *Nature* was accompanied by a commentary by Raine and Gill (2015). The neonicotinoid was applied as the formulation Elado (Bayer) which also contains a pyrethroid β-cyfluthrin; treatment and control sites received a fungicide seed treatment. Wild bees were scarcer in the oilseed rape crop and adjacent uncultivated field borders of treated fields, the analysis controlling for a significant association between flower cover and bee density. Solitary bee nesting success was estimated by placing commercially reared *Osmia bicornis* cocoons in the fields, and observing whether emerging females constructed brood cells in supplied nest traps. None of the female solitary bees in treated fields constructed brood cells, whilst some (but not all) females in 6 of 8 untreated fields did). Bumblebee colony growth and queen production was measured by placing *B. terrestris* colonies adjacent to fields, and honeybee colony performance (number of adult bees) by putting hives at each site. A statistical power analysis indicated that the experimental design would detect a 20% effect on honeybee colony performance, with a probability of 0.8. All pollinator species were shown to feed on oilseed rape, and nectar and pollen collected by honeybees had higher concentrations of clothianidin in treated fields than control fields (10.3 ± 1.3 v 0.1 ± 0.1 ng $g^{-1}$ for nectar; 13.9 ± 1.8 v 0.0 ng $g^{-1}$ for pollen) and similarly for nectar collected by bumblebees (5.4 ± 1.4 v 0.0 ng $g^{-1}$). The non-systemic β-cyfluthrin was not detected.

(A.27) (§40) Authors.

## (g) Consequences of neonicotinoid use

(A.28) (§41) The model, BEEHAVE, is described in Becher *et al.* (2014). They illustrate how it might be used to study neonicotinoid exposure by including the effects of thiamethoxam on forager mortality reported by Henry *et al.* (2012) (as discussed in §35, the doses used are higher than likely to be encountered in the field). They show the consequences of exposure will be greater in poor quality environments and may take several years to become apparent. Bryden *et al.* (2013) describe a model in which sublethal stress affects individual behaviour with knock-on effects for bumblebee colony performance. They show that chronic exposure to sublethal stress can result in colony failure and that model predictions accurately fit data collected from real *B. terresrtris* colonies exposed to imidacloprid under laboratory conditions.

(A.29) Budge *et al.* (2015) provide the data in their paper allowing a reanalysis. They treat nine regions and six time points (with some missing values giving 52 data points). Fitting region and then imidacloprid use to honeybee colony loss, they obtain a significant effect of the insecticide. The effect is weak, explaining 8% of the variation of the data (5% or less if weather covariates are included). If year is added to the model the association with imidacloprid is no longer significant and the variation explained is near zero suggesting that the result is due to a correlation between annual patterns of imidacloprid use (which rise and then fall over this period as neonicotinoids are introduced and then imidacloprid is superseded) and honeybee in-season losses (which show the same general pattern, being highest mid-decade). The critical issue is whether this is causal or correlative. The statistical test that finds the significant association with imidacloprid treats all regions as statistically independent (giving 42 degrees of freedom). If, as is likely, the regional data are affected by unknown common hidden variables (for example widespread weather patterns not captured by the proxies used in the analysis) then the real degrees of freedom will be lower (between 6 and 42) as will the power of the test and the significance of any association.

(A.30) (§42) Kleijn *et al.* (2015) is a meta-analysis of 53 studies of crop pollinators from around the world designed to assess the contribution of different species to crop pollination. They also calculated the economic contribution of wild bees to crop production using the production value method.

(A.31) (§43) Ollerton *et al.* (2014) used a unique database from the Bees, Wasps & Ants Recording Society (BWARS) in the UK to study the temporal distribution and correlates of the 23 recorded extinctions of pollinating bees and wasps. Senapathi *et al.* (2015) repeated historical surveys of land-use and related it to BWARS wild bee and wasp data concluding that bee communities have become less species-rich and more similar over time. These changes were correlated with changes in land-use (and negatively with arable expansion). Kerr *et al.* (2015) analysed 423,000 observation records of 67 bumblebee species over a 110 year period in North America and Europe to build statistical models to test whether range shifts were explained by climate change, land use change or pesticide use. Across both continents, bumblebees were found not to be tracking climate change by expanding their northern limits; however, they were retreating from their southern limits. Shifts in bumblebee ranges were not correlated with changes in land use or pesticide usage, including neonicotinoids, over the same period. Pesticide data was only

available for North America and so was not tested for Europe. The available data used to test whether changes in bumblebee range limits were associated with neonicotinoid use and land-use change were measured at relatively coarse scale. The impacts of neonicotinoids in particular, should they occur, would be expected to affect populations and diversity in subtle ways and at finer scales; dramatic changes in population size could occur within a species distribution range whether or not the overall range is expanding or contracting. Therefore the limitations of the available neonicotinoid data mean that the lack of an effect of neonicotinoid use on range limits should be carefully considered in any inference about neonicotinoid impacts on local abundance, diversity, population trends or individual bee health. Burkle *et al.* (2013) showed degradation of plant-pollinator network structure and loss of species and pollination function at a site in Illinois, USA, was related to global change over 120 years. Scheper *et al.* (2014) analysed pollen loads collected from museum specimens of wild bees, together with wild bee and plant distribution data over time. They concluded that bee declines in the Netherlands were mainly driven by loss of preferred food-plants. Szabo *et al.* (2012) analysed population trends in three species of North American bumblebee and concluded that pesticide use (including neonicotinoids) and habitat loss are unlikely to be major causes of observed declines. Their proxy for insecticide use in their analysis was criticized by Stevens and Jenkins (2013). A revised analysis Colla *et al.* (2013) also found no evidence for neonicotinoid use being correlated with bumblebee decline though the authors caution that the analysis is not definitive. Landscape context may buffer the impact of neonicotinoids on wild bees Park *et al.* (2015). Goulson *et al.* (2015a) review the reasons for the decline of pollinator species and conclude that multiple interacting factors, including pesticides, are responsible. In correspondence arising from the paper the quality of data and need for monitoring was debated Ghazoul (2015), Goulson *et al.* (2015b)

(A.32) (§44) Updated data for Europe (http://ec.europa.eu/food/animals/live_animals/bees/docs/bee-report_en.pdf, http://www.coloss.org/announcements/losses-of-honey-bee-colonies-over-the-2013-14-winter; http://www.coloss.org/announcements/losses-of-honey-bee-colonies-over-the-2014-15-winter-preliminary-results-from-an-international-study) and for the USA Steinhauer *et al.* (2014), Lee *et al.* (2015) and Canada (http://www.capabees.com/ category/extension/overwintering-losses/) are now available. The European data (from before the neonicotinoid restrictions) overall showed the lowest overwintering losses in 2013/2014 since the survey began in 2007.

(A.33) (§45) Unpublished results on the UK Crop Monitor website http://www.cropmonitor.co.uk/wosr/surveys/wosrPestAssLab.cfm?year=2014/2015&season=Spring show comparatively higher levels of beetle pests on oilseed rape in spring 2015, compared to previous years, in some geographical areas but not others. The recent UK oilseed rape study is Budge *et al.* (2015).

(A.34) (§46) Authors.

## References


1. Alburaki M., Boutin S., Mercier P.-L., Loublier Y., Chagnon M., Derome N. 2015 Neonicotinoid-coated *Zea mays* seeds indirectly affect honeybee performance and pathogen susceptibility in field trials. *PLoS One* **10**, e0125790. (doi:10.1371/journal.pone.0125790).
2. Anderson J.C., Dubetz C., Palace V.P. 2015 Neonicotinoids in the Canadian aquatic environment: a literature review on current use products with a focus on fate, exposure, and biological effects. *Science of the Total Environment* **505**, 409-422. (doi:10.1016/j.scitotenv.2014.09.090).
3. Archer C.R., Pirk C.W.W., Wright G.A., Nicolson S.W. 2014 Nutrition affects survival in African honeybees exposed to interacting stressors. *Functional Ecology* **28**, 913-923. (doi:10.1111/1365-2435.12226).
4. Arena M., Sgolastra F. 2014 A meta-analysis comparing the sensitivity of bees to pesticides. *Ecotoxicology* **23**, 324-334. (doi:10.1007/s10646-014-1190-1).
5. Barbieri R.F., Lester P.J., Miller A.S., Ryan K.G. 2013 A neurotoxic pesticide changes the outcome of aggressive interactions between native and invasive ants. *Proceedings of the Royal Society B-Biological Sciences* **280**, 20132157. (doi:10.1098/rspb.2013.2157).
6. Baron G.L., Raine N.E., Brown M.J.F. 2014 Impact of chronic exposure to a pyrethroid pesticide on bumblebees and interactions with a trypanosome parasite. *Journal of Applied Ecology* **51**, 460-469. (doi:10.1111/1365-2664.12205).
7. Becher M.A., Grimm V., Thorbek P., Horn J., Kennedy P.J., Osborne J.L. 2014 BEEHAVE: a systems model of honeybee colony dynamics and foraging to explore multifactorial causes of colony failure. *Journal of Applied Ecology* **51**, 470-482. (doi:Doi 10.1111/1365-2664.12222).
8. Blacquière T., Smagghe G., Van Gestel C.A.M., Mommaerts V. 2012 Neonicotinoids in bees: A review on concentrations, side-effects and risk assessment. *Ecotoxicology* **21**, 973-992. (doi:10.1111/j1439-0418.2011.01648x).
9. Boily M., Sarrasin B., DeBlois C., Aras P., Chagnon M. 2013 Acetylcholinesterase in honey bees (*Apis mellifera*) exposed to neonicotinoids, atrazine and glyphosate: laboratory and field experiments. *Environmental Science and Pollution Research* **20**, 5603-5614. (doi:DOI 10.1007/s11356-013-1568-2).
10. Bonmatin J.M., Giorio C., Girolami V., Goulson D., Kreutzweiser D.P., Krupke C., Liess M., Long E., Marzaro M., Mitchell E.A.D., et al. 2015 Environmental fate and exposure; neonicotinoids and fipronil. *Environmental Science and Pollution Research* **22**, 35-67. (doi:10.1007/s11356-014-3332-7).
11. Bryden J., Gill R.J., Mitton R.A., Raine N.E., Jansen V.A. 2013 Chronic sublethal stress causes bee colony failure. *Ecology Letters* **16**, 1463-1469. (doi:10.1111/ele.12188).
12. Budge G.E., Garthwaite D., Crowe A., Boatman N.D., Delaplane K.S., Brown M.A., Thygesen H.H., Pietravalle S. 2015 Evidence for pollinator cost and farming benefits of neonicotinoid seed coatings on oilseed rape. *Scientific Reports* **5**, 12754. (doi:10.1038/srep12574).
13. Burkle L.A., Marlin J.C., Knight T.M. 2013 Plant-pollinator interactions over 120 Years: loss of species, co-occurrence, and function. *Science* **339**, 1611-1615. (doi:10.1126/science.1232728).
14. Carreck N.L., Ratnieks F.L.W. 2014 The dose makes the poison: have "field realistic" rates of exposure of bees to neonicotinoid insecticides been overestimated in laboratory studies? *Journal of Apicultural Research* **53**, 607-614. (doi:10.3896/Ibra.1.53.5.08).
15. Casida J.E., Durkin K.A. 2013 Neuroactive insecticides: targets, selectivity, resistance, and secondary effects. *Annual Review of Entomology* **58**, 99-117. (doi:10.1146/annurev-ento-120811-153645).
16. Colla S.R., Szabo N.D., Wagner D.L., Gall L.F., Kerr J.T. 2013 Response to Stevens and Jenkins' pesticide impacts on bumblebees: a missing piece. *Conservation Letters* **6**, 215-216. (doi:10.1111/conl.12019).
17. Colombo V., Mohr S., Berghahn R., Pettigrove V.J. 2013 Structural changes in a macrozoobenthos assemblage after imidacloprid pulses in aquatic field-based microcosms. *Archives of Environmental Contamination and Toxicology* **65**, 683-692. (doi:10.1007/s00244-013-9940-2).
18. Cutler G.C., Scott-Dupree C.D. 2014 A field study examining the effects of exposure to neonicotinoid seed-treated corn on commercial bumble bee colonies. *Ecotoxicology* **23**, 1755-1763. (doi:10.1007/s10646-014-1340-5).

19. Cutler G.C., Scott-Dupree C.D., Drexler D.M. 2014a Honey bees, neonicotinoids and bee incident reports: the Canadian situation. *Pest Management Science* **70**, 779-783. (doi:10.1002/ps.3613).
20. Cutler G.C., Scott-Dupree C.D., Sultan M., McFarlane A.D., Brewer L. 2014b A large-scale field study examining effects of exposure to clothianidin seed-treated canola on honey bee colony health, development, and overwintering success. *PeerJ* **2**, e652. (doi:10.7717/peerj.652).
21. Daam M.A., Pereira A.C.S., Silva E., Caetano L., Cerejeira M.J. 2013 Preliminary aquatic risk assessment of imidacloprid after application in an experimental rice plot. *Ecotoxicology and Environmental Safety* **97**, 78-85. (doi:10.1016/j.ecoenv.2013.07.011).
22. de Perre C., Murphy T.M., Lydy M.J. 2015 Fate and effects of clothianidin in fields using conservation practices. *Environmental Toxicology and Chemistry* **34**, 258-265. (doi:10.1002/etc.2800).
23. Dively G.P., Embrey M.S., Kamel A., Hawthorne D.J., Pettis J.S. 2015 Assessment of chronic sublethal effects of imidacloprid on honey bee colony health. *PLoS One* **10**, e0118748. (doi:10.1371/journal.pone.0118748).
24. Doublet V., Labarussias M., de Miranda J.R., Moritz R.F.A., Paxton R.J. 2015 Bees under stress: sublethal doses of a neonicotinoid pesticide and pathogens interact to elevate honey bee mortality across the life cycle. *Environmental Microbiology* **17**, 969-983. (doi:10.1111/1462-2920.12426).
25. Douglas M.R., Rohr J.R., Tooker J.F. 2015 Neonicotinoid insecticide travels through a soil food chain, disrupting biological control of non-target pests and decreasing soya bean yield. *Journal of Applied Ecology* **52**, 250-260. (doi:10.1111/1365-2664.12372).
26. Easton A.H., Goulson D. 2013 The neonicotinoid insecticide imidacloprid repels pollinating flies and beetles at field-realistic concentrations. *PLoS One* **8**, e54819. (doi:10.1371/journal.pone.0054819).
27. Elston C., Thompson H.M., Walters K.F.A. 2013 Sub-lethal effects of thiamethoxam, a neonicotinoid pesticide, and propiconazole, a DMI fungicide, on colony initiation in bumblebee (*Bombus terrestris*) micro-colonies. *Apidologie* **44**, 563-574. (doi:10.1007/s13592-013-0206-9).
28. European Food Safety Authority. 2013a Conclusion on the peer review of the pesticide risk assessment for bees for the active substance clothianidin. *EFSA Journal* **11**, 3066. (doi:10.2903/j.efsa.2013.3066).
29. European Food Safety Authority. 2013b Conclusion on the peer review of the pesticide risk assessment for bees for the active substance imidacloprid. *EFSA Journal* **11**, 3068. (doi:10.2903/j.efsa.2013.3068).
30. European Food Safety Authority. 2013c Conclusion on the peer review of the pesticide risk assessment for bees for the active substance thiamethoxan. *EFSA Journal* **11**, 3067. (doi:10.2903/j.efsa.2012.2668).
31. Fairbrother A., Purdy J., Anderson T., Fell R. 2014 Risks of neonicotinoid insecticides to honeybees. *Environmental Toxicology and Chemistry* **33**, 719-731. (doi: 10.1002/etc.2527).
32. Fauser-Misslin A., Sadd B., Neumann P., Sandrock C. 2014 Influence of combined pesticide and parasite exposure on bumblebee colony traits in the laboratory. *Journal of Applied Ecology* **51**, 450-459. (doi:10.1111/1365-2664.12188).
33. Fischer J., Muller T., Spatz A.K., Greggers U., Grunewald B., Menzel R. 2014 Neonicotinoids interfere with specific components of navigation in honeybees. *PLoS One* **9**, e91364. (doi:10.1371/journal.pone.0091364).
34. Frewin A.J., Schaafsma A.W., Hallett R.H. 2014 Susceptibility of *Aphelinus certus* (Hymenoptera: Aphelinidae) to Neonicotinoid Seed Treatments Used for Soybean Pest Management. *Journal of Economic Entomology* **107**, 1450-1457. (doi:10.1603/Ec13523).
35. Fryday S., Tiede K., Stein J. 2015 Scientific services to support EFSA systematic reviews: Lot 5. Systematic literature review on the neonicotinoids (namely active substances clothianidin, thiamethoxam and imidacloprid) and the risks to bees. *EFSA Supporting Publications* **EN-756**, 1-656.
36. Garbuzov M., Couvillon M.J., Schurch R., Ratnieks F.L.W. 2015 Honey bee dance decoding and pollen-load analysis show limited foraging on spring-flowering oilseed rape, a potential source of neonicotinoid contamination. *Agriculture Ecosystems & Environment* **203**, 62-68. (doi:10.1016/j.agee.2014.12.009).
37. Ghazoul J. 2015 Qualifying pollinator decline evidence. *Science* **348**, 981-982. (doi:10.1126/science.348.6238.981-b).
38. Gibbons D., Morrissey C., Mineau P. 2015 A review of the direct and indirect effects of neonicotinoids and fipronil on vertebrate wildlife. *Environmental Science and Pollution Research* **22**, 103-118. (doi:10.1007/s11356-014-3180-5).
39. Gill R.J., Raine N.E. 2014 Chronic impairment of bumblebee natural foraging behaviour induced by sublethal pesticide exposure. *Functional Ecology* **28**, 1459-1471. (doi:10.1111/1365-2435.12292).
40. Gill R.J., Ramos-Rodriguez O., Raine N.E. 2012 Combined pesticide exposure severely affects individual-and colony-level traits in bees. *Nature* **491**, 105-108. (doi:10.1038/nature11585).
41. Godfray H.C.J., Blacquière T., Field L.M., Hails R.S., Petrokofsky G., Potts S.G., Raine N.E., Vanbergen A.J., McLean A.R. 2014 A restatement of the natural science evidence base concerning neonicotinoid insecticides and insect pollinators. *Proceedings of the Royal Society B-Biological Sciences* **281**, 20140558. (doi:10.1098/rspb.2014.0558).
42. Goulson D. 2015 Neonicotinoids impact bumblebee colony fitness in the field; a reanalysis of the UK's Food & Environment Research Agency 2012 experiment. *PeerJ* **3**, e854. (doi:10.7717/peerj.854).
43. Goulson D., Nicholls E., Botías C., Rotheray E.L. 2015a Bee declines driven by combined stress from parasites, pesticides, and lack of flowers. *Science* **347**, 1255957. (doi:10.1126/science.1255957).
44. Goulson D., Nicholls E., Botías C., Rotheray E.L. 2015b Qualifying pollinator decline evidence: response *Science* **347**, 982. (doi:10.1126/science.348.6238.982).
45. Hallmann C.A., Foppen R.P.B., van Turnhout C.A.M., de Kroon H., Jongejans E. 2014 Declines in insectivorous birds are associated with high neonicotinoid concentrations. *Nature* **511**, 341-343. (doi:10.1038/nature13531).
46. Hatjina F., Papaefthimiou C., Charistos L., Dogaroglu T., Bouga M., Emmanouil C., Arnold G. 2013 Sublethal doses of imidacloprid decreased size of hypopharyngeal glands and respiratory rhythm of honeybees in vivo. *Apidologie* **44**, 467-480. (doi:10.1007/s13592-013-0199-4).
47. Hayasaka D., Suzuki K., Nomura T., Nishiyama M., Nagai T., Sanchez-Bayo F., Goka K. 2013 Comparison of acute toxicity of two neonicotinoid insecticides, imidacloprid and clothianidin, to five cladoceran species. *Journal of Pesticide Science* **38**, 44-47. (doi:10.1584/jpestics.D12-061).
48. Health Canada. 2014 Update on neonicotinoid pesticides and bee health. http://wwwhc-scgcca/cps-spc/alt_formats/pdf/pubs/pest/_fact-fiche/neonicotinoid/neonicotinoid-engpdf.
49. Heimbach U., Stahler M., Schwabe K., Schenke D., Pistoriusl J., Georgiadis P.T. 2014 Emission of pesticides during drilling and deposition in adjacent areas. *Tagungsband: Internationale Fachtagung Zum Forschungsprogramm Uber Den Westlichen Maiswurzelbohrer* **444**, 68-75. (doi:10.5073/jka.2014.444.021).
50. Henry M., Béguin M., Requier F., Rollin O., Odoux J.F., Aupinel P., Aptel J., Tchamitchian S., Decourtye A. 2012 A common pesticide decreases foraging success and survival in honey bees. *Science* **336**, 348-350.
51. Henry M., Bertrand C., Le Feon V., Requier F., Odoux J.F., Aupinel P., Bretagnolle V., Decourtye A. 2014 Pesticide risk assessment in free-ranging bees is weather and landscape dependent. *Nature Communications* **5**, 4359. (doi:10.1038/ncomms5359).
52. Holzschuh A., Dormann C.F., Tscharntke T., Steffan-Dewenter I. 2013 Mass-flowering crops enhance wild bee abundance. *Oecologia* **172**, 477-484. (doi:10.1007/s00442-012-2515-5).
53. Johnson R.M. 2015 Honey bee toxicology. *Annual Review of Entomology* **60**, 415-434. (doi:10.1146/annurev-ento-011613-162005).

54. Jones A., Harrington P., Turnbull G. 2014 Neonicotinoid concentrations in arable soils after seed treatment applications in preceding years. *Pest Management Science* **70**, 1780-1784. (doi:10.1002/ps.3836).

55. Kasiotis K.M., Anagnostopoulos C., Anastasiadou P., Machera K. 2014 Pesticide residues in honeybees, honey and bee pollen by LC-MS/MS screening: reported death incidents in honeybees. *Science of the Total Environment* **485**, 633-642. (doi:10.1016/j.scitotenv.2014.03.042).

56. Kerr J.T., Pindar A., Galpern P., Packer L., Potts S.G., Roberts S.P.M., Rasmont P., Schweiger O., Colla S.R., Richardson L.L., et al. 2015 Climate change impacts on bumblebees converge across continents. *Science* **349**, 177-180. (doi:10.1126/science.aaa7031).

57. Kessler S.C., Tiedeken E.J., Simcock K.L., Derveau S., Mitchell J., Softley S., Stout J.C., Wright G.A. 2015 Bees prefer foods containing neonicotinoid pesticides. *Nature* **521**, 74-76. (doi:10.1038/nature14414).

58. Kleijn D., Winfree R., Bartomeus I., Carvalheiro L.G., Henry M., Isaacs R., Klein A.-M., Kremen C., M'Gonigle L.K., Rader R., et al. 2015 Delivery of crop pollination services is an insufficient argument for wild pollinator conservation. *Nature Communications* **6**, 7414. (doi:10.1038/ncomms8414).

59. Krupke C.H., Long E.Y. 2015 Intersections between neonicotinoid seed treatments and honey bees. *Current Opinion in Insect Science* **10**, 8-13. (doi: 10.1016/j.cois.2015.04.005).

60. Larson J.L., Redmond C.T., Potter D.A. 2013 Assessing insecticide hazard to bumble bees foraging on flowering weeds in treated lawns. *PLoS One* **8**, e66375. (doi:10.1371/journal.pone.0066375).

61. Larson J.L., Redmond C.T., Potter D.A. 2014 Impacts of a neonicotinoid, neonicotinoid-pyrethroid premix, and anthranilic diamide insecticide on four species of turf-inhabiting beneficial insects. *Ecotoxicology* **23**, 252-259. (doi:10.1007/s10646-013-1168-4).

62. Larson J.L., Redmond C.T., Potter D.A. 2015 Mowing mitigates bioactivity of neonicotinoid insecticides in nectar of flowering lawn weeds and turfgrass guttation. *Environmental Toxicology and Chemistry* **34**, 127-132. (doi:10.1002/etc.2768).

63. Lee K.V., Steinhauer N., Rennich K., Wilson M.E., Tarpy D.R., Caron D.M., Rose R., Delaplane K.S., Baylis K., Lengerich E.J., et al. 2015 A national survey of managed honey bee 2013-2014 annual colony losses in the USA. *Apidologie* **46**, 292-305. (doi:10.1007/s13592-015-0356-z).

64. Lu C.S., Warchol K.M., Callahan R.A. 2014 Sub-lethal exposure to neonicotinoids impaired honey bees winterization before proceeding to colony collapse disorder. *Bulletin of Insectology* **67**, 125-130. (doi:None).

65. Lundin O., Rundlöf M., Henrik G.S., Fries I., Bommarco R. 2015 Neonicotinoid insecticides and their impacts on bees: a systematic review of research approaches and identification of knowledge gaps. *PLoS One* **10**, e0136928. (doi:10.1371/journal.pone.0136928).

66. Main A.R., Headley J.V., Peru K.M., Michel N.L., Cessna A.J., Morrissey C.A. 2014 Widespread use and frequent detection of neonicotinoid insecticides in wetlands of Canada's prairie pothole region. *PLoS One* **9**, e101400. (doi:10.1371/journal.pone.0092821).

67. Moffat C., Pacheco J.G., Sharp S., Samson A.J., Bollan K.A., Huang J., Buckland S.T., Connolly C.N. 2015 Chronic exposure to neonicotinoids increases neuronal vulnerability to mitochondrial dysfunction in the bumblebee (*Bombus terrestris*). *FASEB Journal* **29**, 2112-2119. (doi:10.1096/fj.14-267179).

68. Morrissey C.A., Mineau P., Devries J.H., Sanchez-Bayo F., Liess M., Cavallaro M.C., Liber K. 2015 Neonicotinoid contamination of global surface waters and associated risk to aquatic invertebrates: a review. *Environment International* **74**, 291-303. (doi:10.1016/j.envint.2014.10.024).

69. Oliveira R.A., Roat T.C., Carvalho S.M., Malaspina O. 2014 Side-effects of thiamethoxam on the brain and midgut of the africanized honeybee *Apis mellifera* (Hymenoptera: Apidae). *Environmental Toxicology* **29**, 1122-1133. (doi:10.1002/tox.21842).

70. Ollerton J., Erenler H., Edwards M., Crockett R. 2014 Extinctions of aculeate pollinators in Britain and the role of large-scale agricultural changes. *Science* **346**, 1360-1362. (doi:10.1126/science.1257259).

71. Park M.G., Blitzer E.J., Gibbs J., Losey J.E., Danforth B.N. 2015 Negative effects of pesticides on wild bee communities can be buffered by landscape context. *Proceedings of the Royal Society B-Biological Sciences* **282**, 20150299. (doi:10.1098/rspb.2015.0299).

72. Pisa L.W., Amaral-Rogers V., Belzunces L.P., Bonmatin J.M., Downs C.A., Goulson D., Kreutzweiser D.P., Krupke C., Liess M., McField M., et al. 2015 Effects of neonicotinoids and fipronil on non-target invertebrates. *Environmental Science and Pollution Research* **22**, 68-102. (doi:10.1007/s11356-014-3471-x).

73. Pohorecka K., Skubida P., Semkiw P., Miszczak A., Teper D., Sikorski P., Zagibajlo K., Skubida M., Zdanska D., Bober A. 2013 Effects of exposure of honey bee colonies to neonicotinoid seed-treated maize crops. *Journal of Apicultural Science* **57**, 199-208. (doi:10.2478/Jas-2013-0029).

74. Raine N.E., Gill R.J. 2015 Tasteless pesticides affect bees in the field. *Nature* **521**, 38-40. (doi:10.1038/nature14391).

75. Retschnig G., Neumann P., Williams G.R. 2014 Thiacloprid-*Nosema ceranae* interactions in honey bees: host survivorship but not parasite reproduction is dependent on pesticide dose. *Journal of Invertebrate Pathology* **118**, 18-19. (doi:10.1016/j.jip.2014.02.008).

76. Rondeau G., Sanchez-Bayo F., Tennekes H.A., Decourtye A., Ramirez-Romero R., Desneux N. 2014 Delayed and time-cumulative toxicity of imidacloprid in bees, ants and termites. *Scientific Reports* **4**, 5566. (doi:10.1038/srep05566).

77. Rundlöf M., Andersson G.K.S., Bommarco R., Fries I., Hederström V., Herbertsson L., Jonsson O., Klatt B.K., Pedersen T.R., Yourstone J., et al. 2015 Seed coating with a neonicotinoid insecticide negatively affects wild bees. *Nature* **521**, 77-80. (doi:10.1038/nature14420).

78. Samson-Robert O., Labrie G., Chagnon M., Fournier V. 2014 Neonicotinoid-contaminated puddles of water represent a risk of intoxication for honey bees. *PLoS One* **9**, e108443. (doi:10.1371/journal.pone.0108443).

79. Sanchez-Bayo F., Goka K. 2014 Pesticide residues and bees - a risk assessment. *PLoS One* **9**, e94482. (doi:10.1371/journal.pone.0094482).

80. Sandrock C., Tanadini L.G., Pettis J.S., Biesmeijer J.C., Potts S.G., Neumann P. 2014a Sublethal neonicotinoid insecticide exposure reduces solitary bee reproductive success. *Agricultural and Forest Entomology* **16**, 119-128. (doi:10.1111/afe.12041).

81. Sandrock C., Tanadini M., Tanadini L.G., Fauser-Misslin A., Potts S.G., Neumann P. 2014b Impact of chronic neonicotinoid exposure on honeybee colony performance and queen supersedure. *PLoS One* **9**, e103592. (doi:10.1371/journal.pone.0103592).

82. Schaafsma A., Limay-Rios V., Baute T., Smith J., Xue Y.G. 2015 Neonicotinoid insecticide residues in surface water and soil associated with commercial maize (corn) fields in Southwestern Ontario. *PLoS One* **10**, e0118139. (doi:10.1371/journal.pone.0118139).

83. Scheper J., Reemer M., van Kats R., Ozinga W.A., van der Linden G.T.J., Schaminee J.H.J., Siepel H., Kleijn D. 2014 Museum specimens reveal loss of pollen host plants as key factor driving wild bee decline in the Netherlands. *Proceedings of the National Academy of Sciences of the United States of America* **111**, 17552-17557. (doi:10.1073/pnas.1412973111).

84. Scholer J., Krischik V. 2014 Chronic exposure of imidacloprid and clothianidin reduce queen survival, foraging, and nectar storing in colonies of *Bombus impatiens*. *PLoS One* **9**, e91573. (doi:10.1371/journal.pone.0091573).

85. Senapathi D., Carvalheiro L.G., Biesmeijer J.C., Dodson C.A., Evans R.L., McKerchar M., Morton R.D., Moss E.D., Roberts S.P.M., Kunin W.E., et al. 2015 The impact of over 80 years of land cover changes on bee and wasp pollinator communities in England. *Proceedings of the Royal Society B-Biological Sciences* **282**. (doi:10.1098/rspb.2015.0294).

86. Shao X.S., Liu Z.W., Xu X.Y., Li Z., Qian X.H. 2013 Overall status of neonicotinoid insecticides in China: production, application and innovation. *Journal of Pesticide Science* **38**, 1-9. (doi:10.1584/jpestics.D12-037).
87. Simon-Delso N., Amaral-Rogers V., Belzunces L.P., Bonmatin J.M., Chagnon M., Downs C., Furlan L., Gibbons D.W., Giorio C., Girolami V., et al. 2015 Systemic insecticides (neonicotinoids and fipronil): trends, uses, mode of action and metabolites. *Environmental Science and Pollution Research* **22**, 5-34. (doi:10.1007/s11356-014-3470-y).
88. Smit C.E., Posthuma-Doodeman C.J.A.M., van Vlaardingen P.L.A., de Jong F.M.W. 2015 Ecotoxicity of imidacloprid to aquatic organisms: derivation of water quality standards for peak and long-term exposure. *Human and Ecological Risk Assessment* **21**, 1608-1630. (doi:10.1080/10807039.2014.964071).
89. Stanley D.A., Knight M.E., Stout J.C. 2013 Ecological variation in response to mass-glowering oilseed rape and surrounding landscape composition by members of a cryptic bumblebee complexk. *PLoS One* **8**, e65516. (doi:10.1371/journal.pone.0065516).
90. Stanley D.A., Stout J.C. 2014 Pollinator sharing between mass-flowering oilseed rape and co-flowering wild plants: implications for wild plant pollination. *Plant Ecology* **215**, 315-325. (doi:10.1007/s11258-014-0301-7).
91. Steinhauer N.A., Rennich K., Wilson M.E., Caron D.M., Lengerich E.J., Pettis J.S., Rose R., Skinner J.A., Tarpy D.R., Wilkes J.T., et al. 2014 A national survey of managed honey bee 2012-2013 annual colony losses in the USA: results from the Bee Informed Partnership. *Journal of Apicultural Research* **53**, 1-18. (doi:10.3896/Ibra.1.53.1.01).
92. Stevens S.M., Jenkins P.T. 2013 Pesticide impacts on bumblebee decline: a missing piece. *Conservation Letters* **6**, 213-214. (doi:10.1111/conl.12020).
93. Stewart S.D., Lorenz G.M., Catchot A.L., Gore J., Cook D., Skinner J., Mueller T.C., Johnson D.R., Zawislak J., Barber J. 2014 Potential exposure of pollinators to neonicotinoid insecticides from the use of insecticide seed treatments in the mid-southern United States. *Environmental Science & Technology* **48**, 9762-9769. (doi:10.1021/es501657w).
94. Szabo N.D., Colla S.R., Wagner D.L., Gall L.F., Kerr J.T. 2012 Do pathogen spillover, pesticide use, or habitat loss explain recent North American bumblebee declines? *Conservation Letters* **5**, 232-239. (doi:10.1111/j.1755-263X.2012.00234.x).
95. Tan K., Chen W., Dong S., Liu X., Wang Y., Nieh J.C. 2015 A neonicotinoid impairs olfactory learning in Asian honey bees (*Apis cerana*) exposed as larvae or as adults. *Scientific Reports* **5**, 10989. (doi:10.1038/srep10989).
96. Tan K., Chen W.W., Dong S.H., Liu X.W., Wang Y.C., Nieh J.C. 2014 Imidacloprid alters foraging and decreases bee avoidance of predators. *PLoS One* **9**, e102725. (doi:10.1371/journal.pone.0102725).
97. Thompson H.M., Fryday S.L., Harkin S., Milner S. 2014 Potential impacts of synergism in honeybees (*Apis mellifera*) of exposure to neonicotinoids and sprayed fungicides in crops. *Apidologie* **45**, 545-553. (doi:10.1007/s13592-014-0273-6).
98. Tome H.V.V., Barbosa W.F., Martins G.F., Guedes R.N.C. 2015 Spinosad in the native stingless bee *Melipona quadrifasciata:* regrettable non-target toxicity of a bioinsecticide. *Chemosphere* **124**, 103-109. (doi:10.1016/j.chemosphere.2014.11.038).
99. van Dijk T.C., van Staalduinen M.A., van der Sluijs J.P. 2013 Macro-invertebrate decline in surface water polluted with imidacloprid. *PLoS One* **8**, e62374. (doi:10.1371/journal.pone.0062374).
100. Vijver M.G., van den Brink P.J. 2014 Macro-invertebrate decline in surface Water polluted with imidacloprid: a rebuttal and some new analyses. *PLoS One* **9**, e89837. (doi:10.1371/journal.pone.0089837).
101. Williamson S.M., Willis S.J., Wright G.A. 2014 Exposure to neonicotinoids influences the motor function of adult worker honeybees. *Ecotoxicology* **23**, 1409-1418. (doi:10.1007/s10646-014-1283-x).
